# Neuroimaging-based evidence for sympathetic correlation between brain activity and peripheral vasomotion during pain anticipation

**DOI:** 10.1038/s41598-024-53921-4

**Published:** 2024-02-09

**Authors:** Ziqiang Xu, Zu Soh, Yuta Kurota, Yuya Kimura, Harutoyo Hirano, Takafumi Sasaoka, Atsuo Yoshino, Toshio Tsuji

**Affiliations:** 1 Graduate School of Advanced Science and Engineering, Hiroshima University, 1-4-1 Kagamiyama, Higashi-Hiroshima, Hiroshima 739-8527 Japan; 2 Department of Medical Equipment Engineering, Clinical Collaboration Unit, School of Medical Sciences, Fujita Health University, 1-98 Dengakugakubo, Kutsukake-cho, Toyoake, Aichi 470-1192 Japan; 3 Center for Brain, Mind and KANSEI Sciences Research, Hiroshima University, 1-2-3 Kasumi, Minami-ku, Hiroshima, Hiroshima 734-8553 Japan; 4 Department of Psychiatry and Neurosciences, Graduate School of Biomedical and Health Sciences, Hiroshima University, 1-2-3 Kasumi, Minami-ku, Hiroshima, Hiroshima 734-8551 Japan

**Keywords:** Neuroscience, Biomedical engineering

## Abstract

Anticipation of pain engenders anxiety and fear, potentially shaping pain perception and governing bodily responses such as peripheral vasomotion through the sympathetic nervous system (SNS). Sympathetic innervation of vascular tone during pain perception has been quantified using a peripheral arterial stiffness index; however, its innervation role during pain anticipation remains unclear. This paper reports on a neuroimaging-based study designed to investigate the responsivity and attribution of the index at different levels of anticipatory anxiety and pain perception. The index was measured in a functional magnetic resonance imaging experiment that randomly combined three visual anticipation cues and painful stimuli of two intensities. The peripheral and cerebral responses to pain anticipation and perception were quantified to corroborate bodily responsivity, and their temporal correlation was also assessed to identify the response attribution of the index. Contrasting with the high responsivity across levels of pain sensation, a low responsivity of the index across levels of anticipatory anxiety revealed its specificity across pain experiences. Discrepancies between the effects of perception and anticipation were validated across regions and levels of brain activity, providing a brain basis for peripheral response specificity. The index was also characterized by a 1-s lag in both anticipation and perception of pain, implying top-down innervation of the periphery. Our findings suggest that the SNS responds to pain in an emotion-specific and sensation-unbiased manner, thus enabling an early assessment of individual pain perception using this index. This study integrates peripheral and cerebral hemodynamic responses toward a comprehensive understanding of bodily responses to pain.

## Introduction

Pain is commonly defined as an unpleasant sensory and emotional experience associated with, or resembling that associated with, actual and potential tissue damage^1^. The experience of pain leads to the onset of perceptual changes that may begin with early anticipation of pain by integrating contextual information, prompting the body to avoid underlying pain based on previous experience^2^. Pain perception can activate the body’s responses in motor and autonomic dimensions to prepare for and minimize potential injury with the “fight-or-flight” response^3^. Both the central and peripheral nervous systems respond to pain, with the sympathetic nervous system (SNS) in particular functioning to modulate the cardiovascular system to transport the energy resources needed for perception and motor responses^4,5^. Whereas the anticipation of pain is considered an emotional and cognitive attribute of the pain experience, its high correlation with pain modulation, individual differences in pain sensitivity, and even the development of chronic pain syndromes urges us to accurately detect and assess its neural activation status for early medical intervention^5–7^. Physiologically, the anticipation of pain retrieves past pain perceptions from memory and evokes fear and/or anxiety due to possible threats to bodily integrity. Moreover, depending on their duration and level, these evoked emotions could recruit cortical activation and descending activation of the SNS to modulate pain perception and behavior^8–12^. Therefore, these clarified mechanisms provide rich evidence for assessing pain perception and its early anticipation in human neurophysiological and neuroimaging studies.

**Figure 1:**
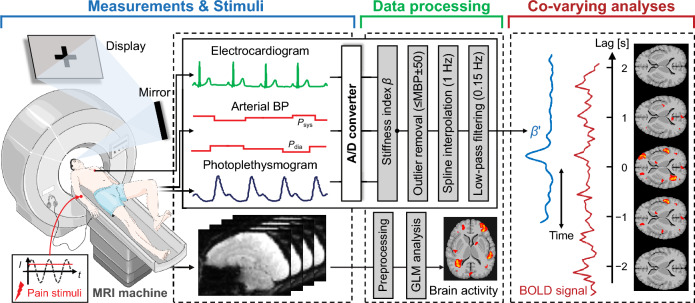
Overview of the method for validating the responsivity of the peripheral arterial stiffness index and brain activity to early anticipation of pain. The method comprises three parts: Measurements and stimuli, data processing, and co-varying analyses. *I*: stimulus current intensity; *t*: time; $P_{\text{sys}}$: systolic blood pressure; $P_{\text{dia}}$: diastolic blood pressure; BP: blood pressure; MBP: mean blood pressure; $\beta$: peripheral arterial stiffness index; $\beta '$: pre-processed index; GLM: general linear model.

The mediating role of the SNS facilitates the noninvasive assessment of anticipatory responses to pain through peripheral measurements^12–15^. Given the integral role of vasomotion in the hemodynamic consequences of sympathetic activation, our research group proposed a noninvasive method to estimate sympathetic modulation of vascular tone using a sympathetic vasomotion assessment index, termed the peripheral arterial stiffness index, which is the inverse of the vascular conductance in the cutaneous vasculature^4,16^. Sympathetic innervation of peripheral arteries normally constricts vascular smooth muscles and stiffens the arterial wall, resulting in a functional elevation in the stiffness of peripheral arteries. Consequently, these functional changes under sympathetic innervation can be quantified to assess the body’s responses to exogenous and endogenous stimuli^17–19^. Our proposed method approximates a nonlinear dynamic relationship between arterial radius and arterial pressure, practically deriving the index through simultaneous measurement of a finger photoplethysmogram (PPG) and continuous arterial blood pressure (ABP). This index has been shown effective in quantitatively assessing subjective pain sensation^20,21^. In addition, a neuroimaging-based study provided evidence that the responsivity of this index following a painful stimulus was positively correlated with the level of brain activity associated with pain perception^22^. However, the experience of pain is subjectively different from the fear and anxiety evoked by pain anticipation^6,23^. Moreover, sympathetic responses in peripheral organs discard the impact of pain on the body in emotional and cognitive dimensions, thus providing only unbiased evidence of human responsivity to stimuli^24^. Therefore, it can be challenging to accurately assess the emotional and cognitive aspects of the body’s anticipatory responses to pain by relying solely on the peripheral arterial stiffness index.

Upon reviewing the clarified mechanisms, the cerebral underpinnings of pain perception and its anticipation have been delineated to identify and dissociate these two processes based on functional neuroimaging methods^2,6,25^. Among these, functional magnetic resonance imaging (fMRI) can noninvasively measure and localize brain activity using the blood oxygen level-dependent (BOLD) signal to detect hemodynamic responses caused by central activation^26^. Utilizing this technique, activated brain regions and their functional connectivity related to pain perception were revealed at the individual level, rendered in the form of a pain matrix mainly comprising anterior cingulate (ACC), insular (INS), prefrontal (PFC), and somatosensory cortices, the thalamus, basal ganglia, cerebellum (Cb), and amygdala^5–7,23,27^. Brain regions activated during pain anticipation, although distinct, are closely related to those involved in pain perception, including the primary somatosensory cortex (S1), ACC, PFC, INS, medial frontal lobe, and Cb^5,6,28^. Further, fMRI also revealed that the level of brain activation associated with pain intensity fluctuates depending on the anticipatory intensity of the pain^28,29^. An fMRI biomarker, based on whole-brain functional connectivity, was proposed to characterize pain-related brain activity and predict the intensity of sustained pain in experimental and clinical settings^30^. In addition, empathy for pain, a physically painless but psychologically anticipated pain process, is mediated by the cognitive and emotional attributes of the pain matrix rather than its sensory attribute, as evidenced by fMRI analyses^25,31^. On the other hand, the combination of neurophysiological and neuroimaging analyses could give rise to converging evidence and provide a more valid basis for developing assessment methods for pain perception and its anticipation. Consequently, central hemodynamic responses depicted by fMRI brain activity should be introduced to enhance our understanding and accurate assessment of bodily responses in anticipation of pain. This would yield neuroimaging-based evidence for corresponding peripheral hemodynamic responses quantified by the peripheral arterial stiffness index.

This paper aims to assess the innervation role of the SNS during pain anticipation by investigating the responsivity of the peripheral arterial stiffness index and its neuroimaging-based evidence. An experiment incorporating anticipation cues and painful stimuli of different intensities was designed to examine and compare peripheral and central hemodynamic responses measured by the index and fMRI time series, respectively. In addition, temporal lags between the two responses were calculated to infer the temporal correlation in a body-wide response to pain perception and its anticipation. This might validate the effectiveness of the index as an early detector of pain experience and further leverage a valid theoretical basis for integrating central and peripheral hemodynamics during pain experience.

## Materials and methods

Figure 1 presents an overview of the study that uses fMRI time series to validate the responsivity of the peripheral arterial stiffness index during pain anticipation; this comprises three parts: measurements and stimuli, data processing, and co-varying analyses. The responsivity of the index and brain activity were evaluated to confirm the consequences of pain anticipation and perception. The covariation in hemodynamic responses between the brain and periphery was analyzed to identify and underpin the attribution of responses of the index to anticipation and perception.

### Participants

We measured the peripheral arterial stiffness index and fMRI time series in 22 healthy young adults (males, age: 22.8 ± 1.2 years [mean ± S.D.]), none of whom had a history of neurological, psychiatric, or chronic pain. All experiments were conducted in accordance with the principles of the Declaration of Helsinki. Informed consent was obtained from all study participants before conducting the experiments, and the study was approved by the Hiroshima University Ethics Committee (Registration number: E-965-5 and E-17-2).

**Figure 2:**
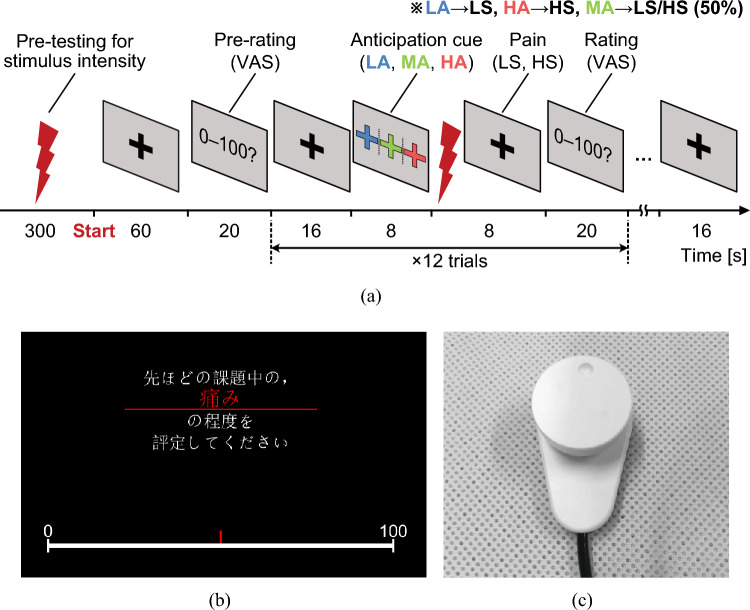
Experimental configurations. (**a**) Experimental protocol for one of the two sessions. VAS: visual analog scale; LA: low anxiety; MA: medial anxiety; HA: high anxiety; LS: low stimulus intensity; HS: high stimulus intensity. (**b**) A rating scale displayed on a screen facing the participants (Japanese text in the image: “Please evaluate the degree of pain during the previous stimulus.”). (**c**) The rotary dial input device for participants to report their subjective sensations.

### Experimental configurations

The experiment includes two consecutive sessions, with the experimental protocol for one session arranged as Fig. 2a. To generate anticipatory anxiety during the experiment, a pre-testing was conducted before the experimental task. The intensity of the stimulus current was set to a value $I_{\text{s}}$ at which the participant verbally reported the pain level as “30” on the visual analog scale (VAS) from 0 to 100 to generate sufficient pain and reduce interindividual differences in pain perception. Each experimental task was initiated with a 60-s rest to relieve participants, followed by the execution of a 20-s pre-rating consisting of four items (pain, anxiety, pleasantness, and unpleasantness). Each trial commenced with a 16-s rest, then an 8-s anticipation cue, an 8-s painful stimulus, and ended with a 20-s rating with the same four items. These steps were repeated randomly a total of 12 times with varying anticipation cues and stimulus intensities. The experimental task ended with a 16-s rest, with a total duration of 1020 s for each session.

All participants were studied in the supine position at a thermoneutral ambient temperature throughout the experiment. In each task, a cue was randomly presented to indicate the probable intensity of a subsequent painful stimulus. Four anticipation cues were designed to induce different levels of anxiety: low stimulus/low anxiety (LS/LA), low stimulus/moderate anxiety (LS/MA), high stimulus/moderate anxiety (HS/MA), and high stimulus/high anxiety (HS/HA). The stimulus currents of 0.5$\times$ and 1.5$\times$ the pre-tested intensity $I_{\text{s}}$ were applied to the participants, corresponding to LS and HS, respectively. During the rest and painful stimuli periods, a “**+**” symbol was displayed to help participants focus and relax. The color of the “**+**” symbols displayed during the anticipation cues corresponded to the level of pain anticipation, where blue indicates LS/LA, green indicates LS/MA or HS/MA with a 50% possibility each, and red indicates HS/HA. In this study, the rating on subjective sensations was gathered using the VAS based on a graduated line mark, with the leftmost 0 representing “no pain” and the rightmost 100 representing “the worst pain imaginable” as shown in Fig. 2b. Rating subjective sensations by moving over the line marker was accomplished using a rotary dial input device as shown in Fig. 2c and visualized by Processing software (Ver.3.0, open project). In subsequent analyses, z-score normalization was applied to the ratings across sessions to minimize inter- and intra-individual differences.

The painful stimulus was generated by an electrocutaneous stimulus system that comprises an isolator (SS-203J, NIHON KOHDEN Corp., Tokyo, Japan), an analog function generation (WF1973, NF Corporation, Kanagawa, Japan), an electric stimulator (SEN-8203, NIHON KOHDEN Corp., Tokyo, Japan) and a surface stimulation electrode (NM-990W, NIHON KOHDEN Corp., Tokyo, Japan). During the painful stimulus experiment, a sinusoidal electrocutaneous stimulus with a frequency of 250 Hz and a predetermined intensity was generated and applied to the participant’s right forearm to evoke pain and activate the SNS.

### Biosignal measurement and processing

#### Biosignal measurement

To analyze the responses of the peripheral arterial stiffness index and brain activity to the early anticipation of pain, electrocardiogram (ECG), noninvasive ABP, and fingertip PPG were measured simultaneously in the MRI environment. A three-lead ECG signal was measured using a BIOPAC ECG100C-MRI amplifier with LEAD110 electrode leads (BIOPAC Systems, Inc., Goleta, CA). ECG was measured to provide cardiac cycle timing for deriving the index beat by beat. The PPG signal from the left index finger was measured using a BIOPAC PPG100C-MRI amplifier with a TSD200-MRI sensor (BIOPAC Systems, Inc., Goleta, CA). The systolic and diastolic blood pressure data were measured at the left middle finger using an MR-compatible device (CareTaker, BIOPAC Systems, Inc., Goleta, CA). All measured biosignals were collected at 1 kHz through a BIOPAC MP 150 modular data acquisition and analysis system (BIOPAC Systems, Inc., Goleta, CA) into a laptop computer and visualized and recorded using AcqKnowledge Data Acquisition and Analysis Software Version 4.2 (BIOPAC Systems, Inc., Goleta, CA).

#### Peripheral arterial stiffness index

As described above, a peripheral arterial stiffness index was proposed to quantify the sympathetic innervation of peripheral arteries by estimating the corresponding changes in arterial diameter and mechanical properties in response to a radial force exerted on the arterial wall. Given the challenge of measuring the continuous arterial blood pressure in the MRI environment, an approximate index was proposed to fulfill the MR compatibility requirements by using only systolic and diastolic blood pressure, defined as^22,32,33^:

1 $$\begin{aligned} \beta = \ln (P_{\text{sys}}/P_{\text{dia}}) / (P_{l \mathrm max}-P_{l \mathrm min}) \end{aligned}$$

where $P_{\text{sys}}$, $P_{\text{dia}}$, $P_{l \mathrm max}$, and $P_{l \mathrm min}$ denote the systolic blood pressure, diastolic blood pressure, and the maximum and minimum values of PPG for each heartbeat, respectively. $\beta$ represents the relative stiffness value of the arterial wall. The high correlation between the primitive and approximate indices has been verified, as has the applicability of the approximate index in the MRI environment^22,34^. Therefore, the approximate index was used to assess the responses of the SNS to the anticipation of pain in this study.

Taking measurement noise into account, outliers in the index $\beta$ would be removed when the corresponding ABP falls outside the range of mean blood pressure ($M\!B\!P\!=\!P_{\text{dia}}+(P_{\text{sys}}-P_\text{dia})/3$)$\pm 50$ mmHg. Since the peripheral arterial stiffness index $\beta$ is calculated beat-to-beat on the basis of the R-peak time of the ECG, cubic spline interpolation is performed within two adjacent R-peak times to construct a continuous curve with a frequency of 1 Hz. Moreover, a 3rd-order Butterworth low-pass filter with a cut-off frequency of 0.15 Hz is subjected to the interpolated results, considering that the respiratory modulation of the cardiovascular system lies in the frequency range of 0.16 to 0.33 Hz and the high-frequency noise in the MRI environment^35,36^. This low-frequency component is considered to be the variation in arterial stiffness, reflecting changes elicited solely by external sensory stimuli and/or mental stress^20,21^. The filtered results are then normalized by their maximum value for the rest period of each trial in the experiment to highlight the variation and reduce inter- and intra-individual differences.

#### Statistical analysis

All statistical tests presented in the manuscript are Brunner–Munzel test with Holm adjustment (significance level: 0.01) unless otherwise noted. Cliff’s delta $\delta$^37^ is presented as a measure of effect size ranging from —1 to +1. The absolute value of $\delta$ indicates the proximity of the two groups, while the sign of $\delta$ denotes whether group A is greater (+) or vice versa (−). The statistical package R, version 4.2.0 (R Foundation for Statistical Computing), was used in this study for statistical analysis.

### fMRI acquisition and preprocessing

#### Data acquisition

A 3-T MRI scanner (MAGNETOM Verio, Siemens Healthcare GmbH, Erlangen, Germany) equipped with a 32-channel head coil was used for the acquisition of MRI data. T2-weighted functional data encompassing the whole brain were acquired with gradient-echo planar imaging (3 × 3 × 4 mm^3^ voxels, slice thickness: 3 mm (without gap), repetition time (TR): 1000 ms, echo time (TE): 30 ms, flip angle: 80°, field of view (FOV): 192 mm, and 42 slices). A high-resolution T1-weighted three-dimensional magnetization-prepared rapid gradient echo (MPRAGE) structural scan (1 × 1 × 1 mm^3^ voxels, slice thickness: 1 mm, TR: 2500 ms, TE: 2.98 ms, flip angle: 9°, FOV: 192 mm, and 176 slices) was performed to normalize and represent the functional data by individuals.

#### Preprocessing

The fMRI data were analyzed using SPM12^38^ and custom MATLAB scripts. The following preprocessing steps were applied to the fMRI data before statistical analysis. The first ten volumes scanned during dummy cycles were discarded to allow for the stabilization of the BOLD signal intensity, resulting in a total of 720 volumes being analyzed in this study. Functional images were then corrected for timing differences in slice acquisition and realigned to the first volume to correct for image intensity outliers resulting from gradient and motion-related artifacts. To further accurately identify areas of brain activation, coregistration was performed between low-resolution functional images and high-resolution structural images. Subsequently, the co-registered functional images for each participant were warped to the Montreal Neurological Institute (MNI) template to match a common anatomical space, reducing inter-individual variations in brain shape and size. Finally, the functional images were smoothed with a Gaussian kernel with a Full-Width at Half-Maximum (FWHM) of 8 mm to suppress noise and enhance the signal.

#### Task analyses

The average brain activity and its group differences in anticipation and perception of pain were estimated and tested using a standard GLM analysis to verify the stimulus consequences and further evidence the attribution of peripheral responses. The first-level GLM analysis was conducted to estimate individual voxel-level brain activations. The 12 task trials in a single session for each participant in the experiment were separately divided and temporally sequenced in series according to the four components of a single run (rest, pain anticipation, painful stimulus, and rating), and each session was analyzed individually. Accordingly, different anticipation cues or painful stimulus intensities were pooled into separate regressors in task analyses. The model also incorporated the six estimated head motion parameters (X, Y, Z, pitch, roll, yaw) as regressors of non-interest, totaling ten regressors for the analysis. To examine brain activity across anticipation cues, 12 contrast images were calculated individually: six for pain anticipation states (HA>LA, HA>MA, MA>LA, LA>HA, MA>HA, and LA>MA) and six for pain perception states (HS/HA>HS/MA, LS/MA>LS/LA, HS/MA>LS/MA, HS/MA>HS/HA, LS/LA>LS/MA, and LS/MA>HS/MA). Second-level (group) analyses were conducted on all analysis results across sessions based on the summary statistic method, testing group differences in average brain activity using one-sample *t*-tests. Significant brain region activation for all participants was accepted with an uncorrected *p*-value of $<\!0.001$ at the voxel level and a cluster-level *p*-value of $<\!0.05$ corrected using FWE (family-wise error).

To clarify the attribution of responses in the peripheral arterial stiffness index, the covariation of responses between brain activity and the index was analyzed according to the temporal correlation^39^. Here, the normalized $\beta _{\text{n}}$ time series of the 12 task trials in each session were also divided to align with the four components of a single trial and sequenced separately alongside the ten regressors described above, for a total of 14 regressors in the first-level model. Moreover, the $\beta _{\text{n}}$ regressors were progressively shifted by 1 s in the range of $-\!$3 to 3 s to conduct a stepwise co-varying analysis, represented by a time lag $t_d$. As earlier, the low- and high-frequency noise was filtered out. Voxel-wise parameter estimates for each covariation were calculated at the individual level, and one-sample *t*-tests were used to identify areas of brain activation that could be covariant with the time-shifted $\beta _{\text{n}}(t+t_d)$ in the group-level analysis. The significant covariation for all participants can be accepted with an uncorrected *p*-value of $<\!0.001$ at the voxel level and a cluster-level *p*-value of $<\!0.05$ corrected using FWE.

**Figure 3:**
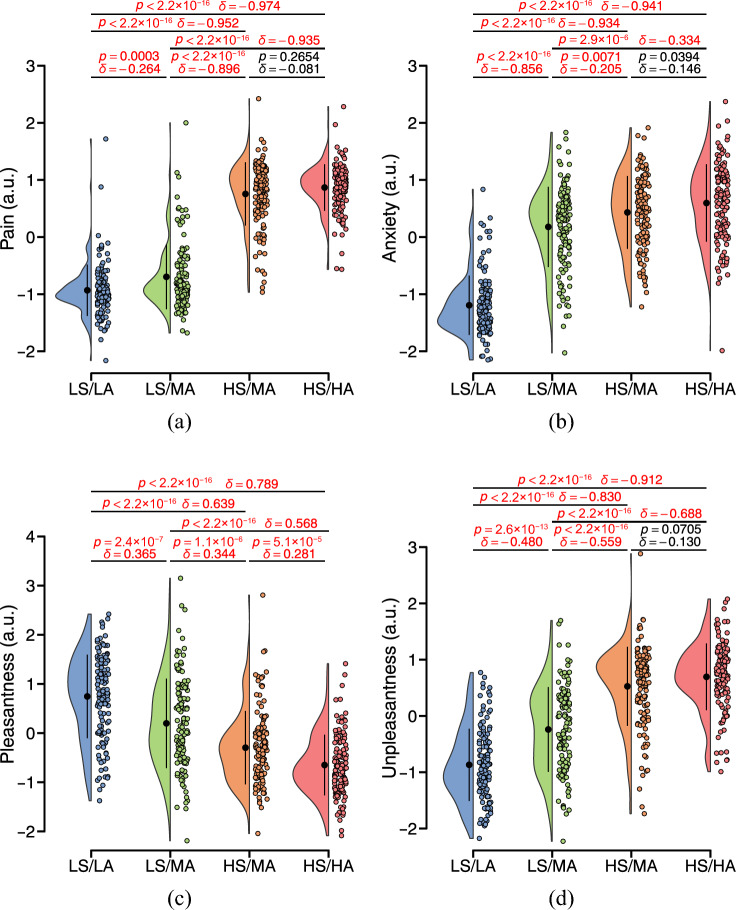
Sensory changes in all participants across different experimental tasks rated by VAS results and normalized by individuals. The black point range lines represent the mean and standard deviation. The statistical test results based on the Cliff’s delta $\delta$ effect size and the Brunner–Munzel test with Holm adjustment are also shown (significance level: 0.01), and significant *p*-values (< 0.01) are in red.

## Results

**Figure 4:**
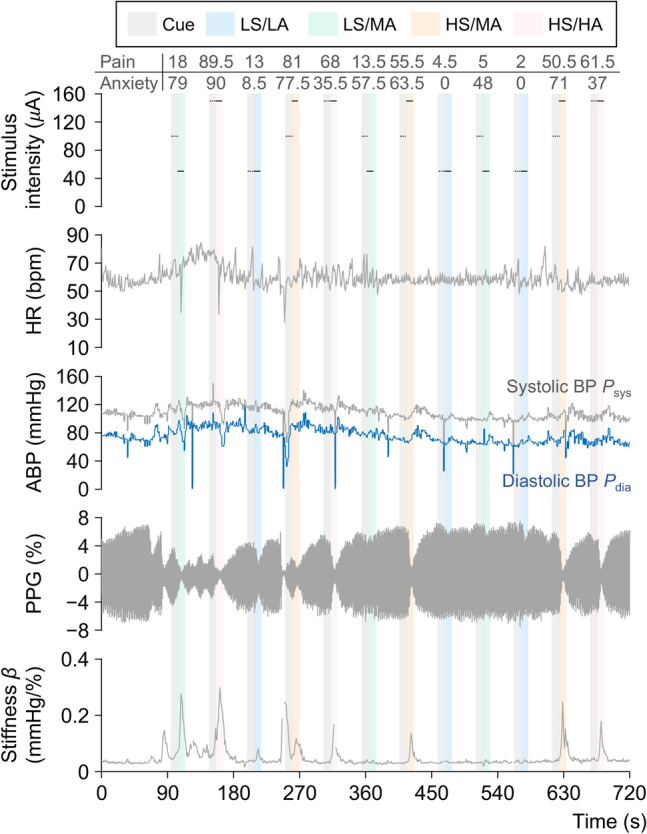
Examples of ratings on pain and anxiety, stimulus intensity, measured heart rate (HR), systolic blood pressure $P_\text{sys}$, diastolic blood pressure $P_{\text{dia}}$, photoplethysmogram (PPG), and peripheral arterial stiffness index $\beta$ from Participant A during session two, respectively. ABP: arterial blood pressure. The black dashed lines represent periods of anticipation cues without physical painful stimuli (no pain).

This study aimed to elucidate the anticipatory responses of the peripheral arterial stiffness index to pain using fMRI analyses. Participants rated pain intensity, anxiety, pleasantness, and unpleasantness following a painful stimulus under each of the four anticipation cues. As expected, painful stimuli of both intensities evoked pain sensations in the population and validated the dose-response relationship between stimulus intensity and pain (Fig. 3a). Comparison of LS/LA and LS/MA yielded a significant difference in pain ratings ($p\!=\!0.0003$, $\delta \!=\!-0.264$), but not between HS/MA and HS/HA ($p\!=\!0.2654$, $\delta \!=\!-0.081$). Consistent with previous research on experimental pain anticipation, anticipation cues before painful stimuli were effective in evoking anxiety in individuals and also exhibited a steep dose-response relationship between LS/LA and the other three levels in terms of anxiety ratings (Fig. 3b, $p\!<\!2.2\!\times \!10^{-16}$, $\delta \!=\!-0.856, -0.934$, and $-0.941$). A comparison between LS/MA and HS/MA yielded a significant difference in anxiety ratings ($p\!=\!0.0071$, $\delta \!=\!-0.205$), but not between HS/MA and HS/HA ($p\!=\!0.0394$, $\delta \!=\!-0.146$). The opposite patterns of results were found for pleasantness and unpleasantness ratings (Fig. 3c and d), which respectively decreased and increased with increasing levels of anticipation cues in an approximately linear dose-response relationship. The pleasantness and unpleasantness ratings were significantly different across anticipation cues, except for the comparison between HS/MA and HS/HA in unpleasantness ratings ($p\!=\!0.0705$, $\delta \!=\!-0.130$).

**Figure 5:**
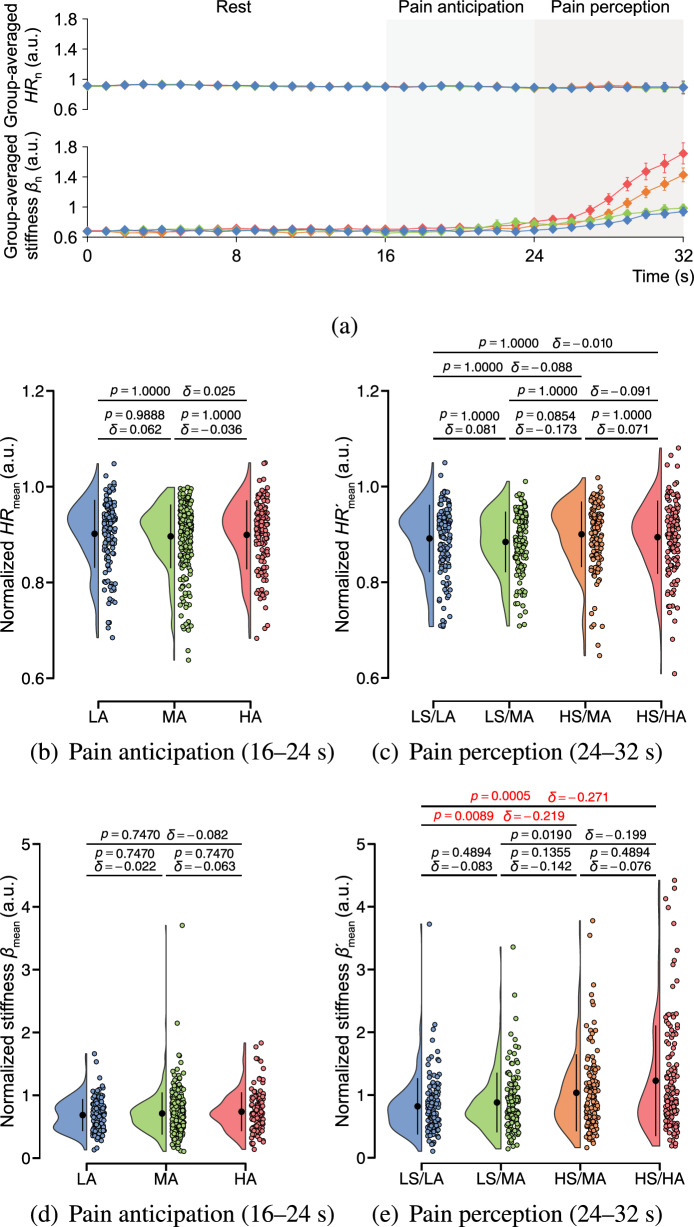
Normalized $H\!R_{\text{n}}$ and $\beta _{\text{n}}$ for all participants in the experiment. (**a**) Group-averaged results across individuals. Values are mean ± SEM. (**b**–**e**) Mean values across anticipation cues. The black point range lines represent the mean and standard deviation. The statistical test results based on the Cliff’s delta $\delta$ effect size and the Brunner–Munzel test with Holm adjustment are also shown (significance level: 0.01), and significant *p*-values (< 0.01) are in red.

**Figure 6:**
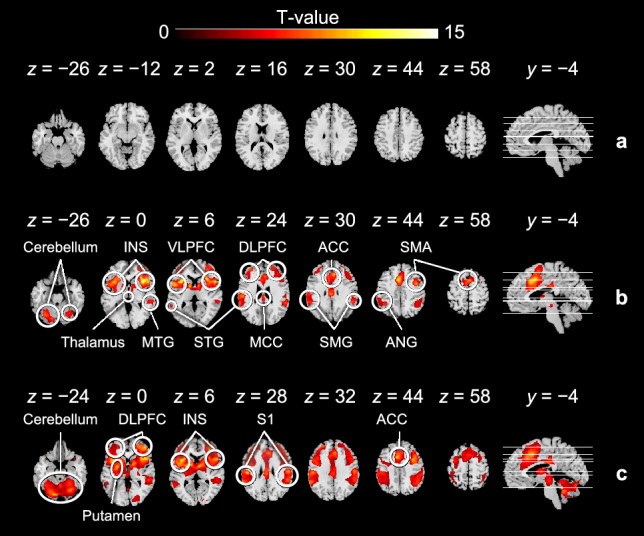
Areas of brain activation for all participants. (**a**) At rest. (**b**) Period of pain anticipation. (**c**) Period of pain perception. Here, analysis results across sessions covered all anticipation cues or painful stimulus intensities. The significant activation of brain regions for all participants can be accepted with an uncorrected *p*-value of $<\!0.001$ at the voxel level and a cluster-level *p*-value of $<\!0.05$ corrected using FWE.

Next, peripheral hemodynamic responses during the anticipation of pain and subsequent painful stimuli were investigated based on a panoply of measured and calculated biosignals shown as examples of time-series waveforms in Fig. 4. Peripheral activation was quantified using the peripheral arterial stiffness index based on the rise in blood pressure and the decline in PPG amplitude, which may be attributed to SNS activation triggered by corresponding emotional and sensory changes. Following several successive experimental runs, sensory adaptation and refractoriness of peripheral responses in the participant were observed at the lower levels of anticipation cues during the 450–600 s period. HR, a commonly used index reflecting psychological status, was also examined in this study to assess cardiac responses to emotional and sensory changes. However, no significant increase was observed across individuals or anticipation cues as shown in Fig. 5a. A slight increase in the group average of the normalized index $\beta _{\text{n}}$ was found during pain anticipation, and a more pronounced increase was found during pain perception, particularly for the anticipation cues of HS/HA and HS/MA. To expose the responsivity of the index, the normalized $\beta _{\text{n}}$ and $H\!R_{\text{n}}$ during the anticipation of pain and subsequent painful stimuli were respectively averaged and investigated across anticipation cues as shown in Fig. 5b–e. No significant differences in $H\!R_{\text{mean}}$ were identified across the various anticipation cues from pain anticipation to pain perception ($p\!>\!0.01$, $|\delta |\!<\!0.1$). Despite no significant differences in $\beta _{\text{mean}}$ were found among the different anticipation cues during pain anticipation (Fig. 5d), there was a significant elevation in $\beta _\text{mean}$ when comparing LS/LA to HS/MA and HS/HA (Fig. 5e, $p\!=\!0.0089$, $\delta =-0.219$; $p\!=\!0.0005$, $\delta \!=\!-0.271$), but only a minor elevation between LS/MA and HS/MA and HS/HA ($p\!=\!0.1355$, $\delta =-0.142$; $p\!=\!0.0190$, $\delta \!=\!-0.199$).

**Figure 7:**
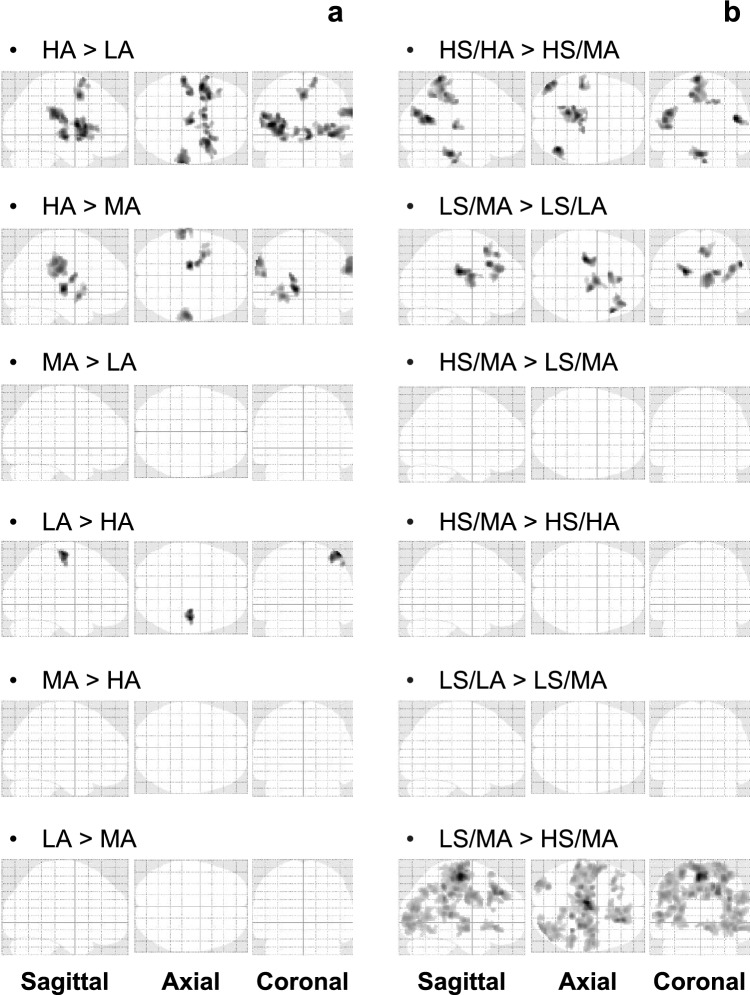
Group analysis results for the contrasts across anticipation cues in both sessions for all participants. (**a**) Pain anticipation. (**b**) Pain perception. The significant activation of brain regions for all participants can be accepted with an uncorrected *p*-value of $<\!0.001$ at the voxel level and a cluster-level *p*-value of $<\!0.05$ corrected using FWE.

Next, a whole-brain voxel-wise GLM analysis was performed to assess the activation of brain regions associated with pain and anxiety due to emotional and sensory stimuli, thereby identifying the attribution of peripheral hemodynamic responses to the anticipation of pain and subsequent painful stimuli. Participants were calm at rest and exhibited no significant activation in the brain as shown in Fig. 6a. A set of brain regions exhibited activation during pain anticipation, including the INS, ventrolateral prefrontal cortex (VLPFC), dorsolateral prefrontal cortex (DLPFC), supplementary motor area (SMA), thalamus, ACC, medial cingulate cortex (MCC), superior temporal gyrus (STG), angular gyrus (AnG), supramarginal gyrus (SMG), middle temporal gyrus (MTG), and Cb (Fig. 6b and Supplementary Table 1). The activation during painful stimuli was also observed in a set of brain regions, including the S1, INS, ACC, DLPFC, putamen, and Cb (Fig. 6c and Supplementary Table 1). All voxel-wise results reported are significant across individuals at a *p*-value of $<\!0.001$ voxel uncorrected and a *p*-value of $<\!0.05$ cluster FWE-corrected. These activated brain regions have been previously reported to be associated with pain anticipation (INS, ACC^5,6,28^), pain perception (S1, ACC, INS, and prefrontal cortex^5,6,40^), and sympathetic activation (DLPFC^41,42^), preliminarily attributing the peripheral hemodynamic responses to the descending activation of the SNS elicited by anticipatory anxiety and pain perception.

Given the observed differences in the responsivity of the peripheral arterial stiffness index, the responsivity of brain activity across anticipation cues was subsequently investigated by contrasting each anticipation cue against another at the voxel level. For the contrast HA>LA, the AIC, putamen, Cd, S1, thalamus, SMA, and VLPFC were significantly activated. For HA>MA, significant activation was found in the AIC, thalamus, S1, and SMG, suggesting a positive association between levels of brain activity and anxiety due to differences in AIC activation^43^. For LA>HA, the SMA and S1 were significantly activated, hinting at a modulation of pain perception and the involvement of bodily defense actions during pain anticipation^44^. Conversely, no significant activation was found for contrasts MA>LA, MA>HA, and LA>MA, as shown in Fig. 7a. As for pain perception, significant activation was observed for the contrast HS/HA>HS/MA in the AnG, SPL, dPCC, M1, Cb, and visual association cortex, indicating differences in sensory input and arousing attention at levels of anxiety under the same stimulus intensity^45^. For LS/MA>LS/LA, regions related to subjective pain experience, including the VLPFC, thalamus, DLPFC, Cd, and SMA, were significantly activated, suggesting a modulatory role of anxiety on pain perception. For LS/MA>HS/MA, the SMA, S1, DLPFC, VLPFC, ACC, and Cd were significantly activated under the same anticipation cue. This activation of brain regions linked to reassurance and pleasant emotions was likely due to the actual stimulus intensity being lower than anticipated^44,46^. Furthermore, there was no significant activation for contrasts HS/MA>LS/MA, HS/MA>HS/HA, and LS/LA>LS/MA as shown in Fig. 7b. Thus, brain activity exhibits response specificity across anticipation cues that elicit different degrees of anxiety and pain.

**Figure 8:**
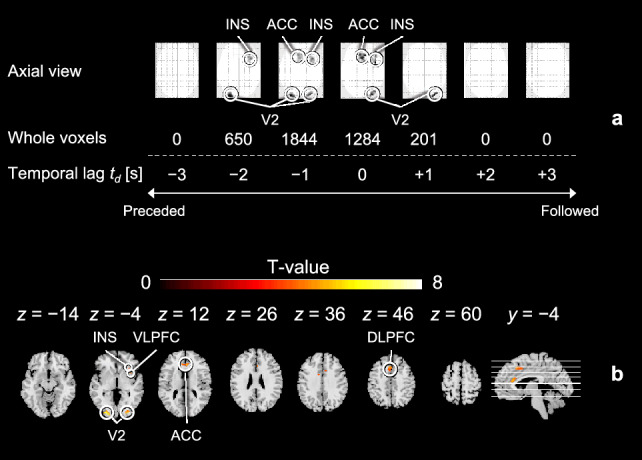
Temporal correlation of the peripheral and cerebral hemodynamic responses during pain anticipation across sessions for all participants. (**a**) The number of significantly activated voxels and their corresponding brain regions when the temporal lag $t_d$ between the BOLD signal and normalized stiffness index $\beta _\text{n}$ ranged from $-3$ s to $+3$ s. “0 s” indicates that the cerebral hemodynamic response should covary with that in the peripheral sites. “Preceded” indicates the cerebral hemodynamic response should be faster than that in the peripheral sites, while “Followed” is the opposite. (**b**) Axial and sagittal views of the brain when $t_d=-1$ s.

**Figure 9:**
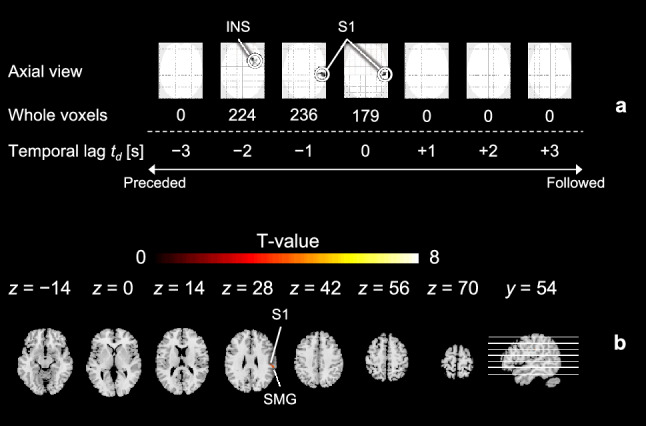
Temporal correlation of the peripheral and cerebral hemodynamic responses during pain perception across sessions for all participants. (**a**) The number of significantly activated voxels and their corresponding brain regions when the temporal lag $t_d$ between the BOLD signal and normalized stiffness index $\beta _\text{n}$ ranged from $-3$ s to $+3$ s. “0 s” indicates that the cerebral hemodynamic response should covary with that in the peripheral sites. “Preceded” indicates the cerebral hemodynamic response should be faster than that in the peripheral sites, while “Followed” is the opposite. (**b**) Axial and sagittal views of the brain when $t_d=-1$ s.

Finally, time-series waveforms of the peripheral arterial stiffness index were incorporated as covariates of interest in the voxel-wise GLM analysis to determine the attribution of its responses in terms of temporal correlation between cerebral and peripheral hemodynamic responses. Cerebral hemodynamic responses to the anticipation of pain were found to be significantly covariant with peripheral hemodynamic responses as shown in Fig. 8a, reaching its strongest covariation (1844 voxels) at $t_d\!=\!-\!$1 s out of a significant time lag of $-\!$2 to 1 s. As shown in Fig. 8b, the co-activated brain regions included the secondary visual cortex (V2), INS, VLPFC, DLPFC, and ACC, which were associated with the anticipation of pain induced by visual transmission, providing evidence that peripheral hemodynamic arousal could be a manifestation of cerebral activation due to pain anticipation. Similarly, the strongest covariation (236 voxels) between the cerebral and peripheral hemodynamic responses to pain perception was observed at $t_d\!=\!-1$ s out of a significant time lag of $-\!$2 to 0 s, as shown in Fig. 9a. The co-activated brain regions included the S1 and SMG involved in pain perception as shown in Fig. 9b. This evidences a distinct central modulation of the cardiovascular system during painful stimuli, differing from that in pain anticipation. Together, these findings suggest that peripheral hemodynamic responses characterized by the index are centrally modulated differentially during the anticipation and perception of pain and exhibit a 1-s lag over the cerebral hemodynamic response.

## Discussion

In this study, the responsivity of the peripheral arterial stiffness index in the anticipation and perception of pain was investigated with fMRI-based evidence. To this end, four anticipation cues were applied to healthy human participants with multiple biosignals measured simultaneously in an MRI environment. Subjective ratings suggested that anticipation of pain influenced pain perception only at the low stimulus intensity, whereas painful stimulus intensity was the primary determinant of peripheral hemodynamic responses. Neuroimaging analyses of fMRI data revealed that the brain was activated in anticipation and perception and exhibited a higher specificity in response to different anticipation cues. Central to the fMRI-based evidence was a co-varying analysis of cerebral and peripheral hemodynamic responses, which enabled the translation of the two responses from their temporal correlation to the underlying innervation mechanisms, yielding direct evidence for a sympathetic correlation between the brain and the periphery^47^.

### Anticipation effects on pain perception

Anticipation cues intervened in pain perception by recruiting anxiety under low-intensity stimuli, and its anticipation effects failed under high-intensity stimuli. Uncertainty about the intensity of an impending painful stimulus (MA) triggered comparable levels of anxiety as certainty about a high-intensity stimulus, suggesting a mediating role for predictability in shaping pain perception by harnessing potential anxiety or fear^48–51^. The underlying dose-response relationship between anticipation cues and pain intensity indicates a positive contribution of moderate anticipatory activation in eliciting anxiety and hyperalgesia^8,52^. Furthermore, a negative effect on participants’ cognitive appraisal of impending pain was discerned based on the approximately linear dose-response relationships involving pleasantness and unpleasantness ratings. This finding forwards the bidirectional relationship between pain and cognition and indicates the potential for differential modulation of brain activity and subsequent peripheral responses in anticipation of pain^25,53,54^. Therefore, the designed anticipation cues enabled different anticipatory effects on pain perception, providing a valid experimental basis for subsequent analyses of cerebral and peripheral hemodynamic responsivity.

#### Bodily responsivity in anticipation and perception of pain

The peripheral hemodynamic responses were aroused and modulated through different neural pathways in the anticipation and perception of pain, thus exhibiting different responsivities characterized by the peripheral arterial stiffness index. Cortical cardiovascular modulation is directly involved in anticipation of pain without sensory afferent signals, whereas medullary modulation dominates pain perception following pain afferents^11,55^. Both types of central regulation involve the mediated activation of the SNS and its tonic constricting effect on the vascular smooth muscle cells of the peripheral arterial wall, leading to the resulting rise in the index. The nerve conduction velocities of sympathetic vasoconstrictor and sudomotor bursts recorded from the tibial nerve are approximately 0.76 and 0.95 m/s, and changes in skin conductance due to sweating in the hands were reported an onset latency of 1.3–1.5 s^56,57^. In addition, the BOLD response typically has a delay of 1–2 s to the stimulus, peaking around 4–6 s after stimulus onset^58^. Overall, a top-down innervation of the periphery can be inferred using the peripheral arterial stiffness index based on the 1-s lag between peripheral and cerebral hemodynamic responses^59^.

Concurrently, discrepancies in the response specificity of the peripheral arterial stiffness index between periods of pain anticipation and pain perception were also revealed based on its responses across anticipation cues. Whereas different brain regions activated across periods and anticipation cues lead to different responsivity of the index, it is also plausible for functional connectivity between relevant regions to contribute to the responsivity^5^. There is some evidence that functional connectivity for pain anticipation extends from higher-order to sensory brain regions, and the opposite is true for pain perception, which is consistent with the direction of central innervation of the SNS. More importantly, pain anticipation has been demonstrated to primarily affect interregional functional connectivity rather than altering local brain activity, whereas pain perception primarily impacts local brain activity based on stimulus intensity^2^. Hence, the absence of significant variation in local brain activity may contribute to the low responsivity of the index in anticipation of pain. However, this evidence does not account for the potential impacts of uncertainty about stimulus intensity and the resulting anxiety on brain activity. Our findings may capture the mediating role of predictability and its subsequent effect on brain activity levels and regions during pain anticipation, which may minimally impact peripheral hemodynamic responses. Combined with the fact that the responsivity of the index coincides with the responsivity of brain activity in response to painful stimuli^22^, the validity of the index in the early detection and assessment of pain experience is clarified on the basis of neuroimaging evidence.

#### Underlying mechanisms bridge cerebral and peripheral responses

Our findings on the temporal correlation analyses suggest that the link between cerebral and peripheral hemodynamics may lie in the 1-s lag observed through the peripheral arterial stiffness index. A few studies have shown evidence that functional interactions in the brain could be extended to the heart in a bidirectional manner through sympathovagal control^60^. Furthermore, this bidirectional mechanism has also been confirmed to maintain fear balance across fear levels by stressing the upward input of the vagus nerve to the INS^61^. Thus, the mediating role of the autonomic nervous system may underline a similar bidirectional relationship of neural control between brain activity and peripheral hemodynamics in the anticipation and perception of pain. Although there is consensus on the lack of direct innervation of the peripheral arteries by the vagus nerve, the withdrawal of sympathetic tone may be attributed to upward signaling of vagal activation, which could modulate cerebrovascular tone and further interact with brain activity^59,62^. Therefore, the relationship between vagal innervation and this index should be further clarified on the basis of cerebral and peripheral hemodynamics. Moreover, the duration and level of SNS activation are related to the bodily modulation of pain^10^, which can be quantified by the index. Therefore, this evidence sets the stage for future work with the specific aim of incorporating peripheral hemodynamics modeled by the index into the analysis of interregional functional connectivity between the brain and periphery in order to investigate the bottom-up influence on brain activity in the anticipation and perception of pain by establishing a global BOLD signal.

#### Limitations

Considering the gender differences in pain perception and anxiety^63^, the results obtained in this study should be further validated in female participants. The low levels of sympathetic activation associated with the experimental protocol were elicited under low intensity and short duration of the stimulus. Consequently, the underlying cause of the low responsivity of the peripheral arterial stiffness index may be the low degree of anxiety in anticipation of pain. Then, the temporal lag between peripheral and cerebral hemodynamic responses cannot be accurately interpreted due to the lack of direct measurements of neural activity in the SNS. Moreover, although the two sessions were analyzed separately, further studies are needed to investigate adaptations in participants’ brain activity, arterial stiffness, or reported sensations between sessions. Finally, the index quantifies functional changes in arterial stiffness in response to sympathetic activation due to stimuli or triggers. Therefore, its applicability to pathological subjects with different degrees of arterial stiffness (e.g., hypertensive patients) requires further evaluation.

## Conclusions

This study leverages neuroimaging-based evidence to elucidate the response specificity of the peripheral arterial stiffness index to pain anticipation and perception, suggesting a sympathetic correlation between brain activity and peripheral vasomotion. The low responsivity during pain anticipation, high responsivity during pain perception, and the lagging characteristics of the index suggest that pain experiences of varying emotions and degrees lead to different characteristic patterning of central innervation and resulting differences in SNS activation. These results provide parallel neuroimaging and neurophysiological insights into cerebral and peripheral mechanisms of pain and its anticipation, fostering an unbiased approach to perceiving and assessing individual pain experiences. Furthermore, this study justifies efforts to unravel the link between the brain and the periphery, delivering a more comprehensive understanding of bodily responses to stimuli and informing the development of more clinically effective interventions for pain alleviation.

To refine the global hemodynamic linkage, further analyses of the interregional functional connectivity of pain anticipation and perception are necessary by incorporating the peripheral arterial stiffness index. It is also imperative to evaluate and synopsize the response characteristics of the index to remove the need for direct measurement of neural activity, thus providing firmer theoretical evidence for global hemodynamics. Furthermore, clinical patients should be considered in future studies to explore relevant pathologic changes and to provide further information about potential starting points for pain interventions.

### Supplementary Information

Supplementary Tables.

## Data Availability

The datasets generated and analyzed during the current study are not publicly available but are available from the corresponding author on reasonable request.
