# Supplementary material for: Neuroimaging-based evidence for sympathetic correlation between brain activity and peripheral vasomotion during pain anticipation
Source: Sci Rep. 2024 Feb 9;14:3383. doi: 10.1038/s41598-024-53921-4 (PMC10858222; doi:10.1038/s41598-024-53921-4)
Supplement: Supplementary file 1 — Supplementary Tables. [file 41598_2024_53921_MOESM1_ESM.pdf]

Supplementary Information for

Neuroimaging-based Evidence for Sympathetic Correlation between Brain Activity and Peripheral Vasomotion during Pain Anticipation

Ziqiang Xu, Zu Soh*, Yuta Kurota, Yuya Kimura,
Harutoyo Hirano, Takafumi Sasaoka, Atsuo Yoshino, Toshio Tsuji*

*Corresponding author(s). E-mail(s): sozu@hiroshima-u.ac.jp;
tsuji-c@bsys.hiroshima-u.ac.jp;

The PDF file includes:

Supplementary Information
This file contains Supplementary results of MNI coordinates for each neuroimaging analysis in this study.
Table S1. MNI coordinates of the activated clusters during rest, period of pain anticipation, and period of pain perception.
Table S2. MNI coordinates of the contrasts across anticipation cues for all participants during pain anticipation.
Table S3. MNI coordinates of the contrasts across anticipation cues for all participants during pain perception.
Table S4. MNI coordinates of the activated clusters in temporal causality analysis of the peripheral and cerebral hemodynamic responses during pain anticipation.
Table S5. MNI coordinates of the activated clusters in temporal causality analysis of the peripheral and cerebral hemodynamic responses during pain perception.

Supplementary Table S1 MNI coordinates of the activated clusters during rest, period of pain anticipation, and period of pain perception. The significant activation of brain regions for all participants can be accepted with an uncorrected p-value of < 0.001 at the voxel level and a cluster-level p-value of < 0.05 corrected using FWE. BA: Brodmann's area.

**Region**	**BA**	X**[mm]**	Y**[mm]**	Z**[mm]**	Z**-value**	**Voxels**
A. Rest						
No significant clusters						
B. Pain anticipation						
INS						
AIC	13	40	20	0	6.96	7609
COp	13	36	6	10	5.25	
VLPFC						
IFGOp	44	54	16	6	6.25	
FOp	47	50	24	−2	6.20	
IFGOr	47	36	30	−4	6.17	
SMA						
PrG	6	42	4	46	5.98	
DLPFC						
MFG	10	46	50	0	5.30	
INS						
FOp	13	−36	16	6	6.78	7250
COp	13	−36	2	12	5.25	
VLPFC						
IFGOp	44	−56	10	10	6.46	
DLPFC						
MFG	10	−34	52	24	5.33	
IFGTr	46	−42	40	6	4.86	
Thalamus						
Thalamus	/	12	−6	8	4.69	
ACC						
SMC	32	−6	14	44	6.53	
ACgG	24	2	24	20	4.71	
DLPFC						
SFGM	8	8	30	30	6.30	
SFG	10	−14	10	66	5.46	
SMA						
SMC	6	8	14	56	5.79	
SFG	6	6	20	66	5.16	
STG						
PT	22	−60	−40	20	5.12	3120
AnG						
SMG	39	−64	−44	28	5.00	
STG	39	−62	−48	12	4.38	
MTG	39	−60	−56	10	4.27	
AnG	39	−50	−58	48	3.73	
MCC						
MCgG	/	0	−22	28	5.41	641
Cb						
Cb	/	−16	−78	−26	5.41	1881
SMG						
SMG	40	56	−40	28	5.31	3096
STG	22	62	−42	20	4.93	
COp	40	60	−12	16	3.62	
MTG						
MTG	21	62	−34	2	4.83	
AnG						
AnG	39	40	−58	48	4.30	
Cb						
Cb	/	24	−66	−26	4.68	604

continued on next page

continued from previous page

Region	BA	X[mm]	Y[mm]	Z[mm]	Z-value	Voxels
C. Pain perception						
S1						
PoG	1	−50	−22	28	6.43	47666
INS						
FOp	13	−40	12	6	6.37	
FOp	13	44	22	−4	6.26	
AIC	13	40	20	−2	6.18	
ACC						
SMC	32	−6	14	44	6.31	
Pu						
Pu	/	−30	−6	−2	6.24	
DLPFC						
MFG	10	46	48	0	6.18	
Cb						
Cb	/	−16	−80	−24	6.19	12970
VER VIII-X	/	0	−56	−32	5.74	
Cb	/	26	−68	−24	5.49	

AIC: anterior insula cortex; ACC: anterior cingulate cortex; IFGOp: opercular part of the inferior frontal gyrus; FOp: frontal operculum; IFGOr: orbital part of the inferior frontal gyrus; PrG: precentral gyrus; MFG: middle frontal gyrus; COp: central operculum; PIns: posterior insula; IFGTr: triangular part of the inferior frontal gyrus; VDC: Ventral dorsal caudate; SMC: supplementary motor cortex; SFGM: superior frontal gyrus medial segment; SFG: superior frontal gyrus; ACgG: anterior cingulate gyrus; PT: planum temporale; SMG: supramarginal gyrus; STG: superior temporal gyrus; MTG: middle temporal gyrus; AnG: angular gyrus; MCgG: middle cingulate gyrus; PoG: postcentral gyrus; Cb: cerebellum; VER: Cerebellar Vermal Lobules VIII-X; Pu: putamen.

Supplementary Table S2 MNI coordinates of the contrasts across anticipation cues for all participants during pain anticipation. The significant activation of brain regions for all participants can be accepted with an uncorrected p-value of < 0.001 at the voxel level and a cluster-level p-value of < 0.05 corrected using FWE. BA: Brodmann's area.

**Region**	**BA**	X**[mm]**	Y**[mm]**	Z**[mm]**	Z**-value**	**Voxels**
A. HA>LA						
SMA						
PrG	6	−44	0	12	4.64	950
INS						
AIC	13	−30	12	8	4.51	
INS	/	−32	10	2	4.39	
INS	/	−34	0	18	3.53	
COp	44	−40	6	6	4.22	
VLPFC						
IFGOp	44	−58	8	12	3.91	
IFGTr	45	−38	26	6	3.70	
Pu						
Pu	/	−28	2	14	4.13	
Pu	/	−28	2	−8	4.03	
GP						
GP	/	−10	4	2	3.87	
Thalamus						
VDC	/	6	−16	−6	4.43	218
Thalamus	/	6	−18	2	4.18	
VDC	/	−6	−14	−10	3.45	
INS						
AIC	13	38	8	−2	4.51	767
Pu	13	30	14	8	4.03	
AIC	13	38	0	10	3.97	
AIC	13	32	20	8	3.86	
Pu						
Pu	/	22	8	2	3.96	
VLPFC						
IFGOr	47	52	20	−6	3.57	
IFGOp	44	60	16	8	3.42	
Thalamus						
Thalamus	/	14	−4	12	3.35	
SMG						
SMG	40	66	−30	26	4.35	432
POp	40	52	−26	24	3.82	
SMG						
STG	40	−54	−28	22	4.35	330
INS						
INS	/	−36	−20	22	4.16	
S1						
COp	1	−44	−20	16	3.77	
SMA						
SMC	6	−2	4	50	4.31	297
SFG	6	14	8	70	3.88	
SFG	6	12	6	64	3.81	
SMC	6	−6	−2	56	3.53	
SMA	6	10	16	62	3.50	

continued on next page

continued from previous page

**Region**	**BA**	X**[mm]**	Y**[mm]**	Z**[mm]**	Z**-value**	**Voxels**
B. HA>MA						
Thalamus						
Thalamus	/	−12	−18	2	5.13	256
Thalamus	/	−16	−6	16	4.48	
SMG						
SMG	40	60	−22	28	4.53	620
SMG	40	62	−22	42	4.02	
SMG	40	64	−32	34	3.74	
STG	22	66	−30	18	3.44	
SMG						
SMG	40	−64	−28	38	4.42	503
POp	40	−54	−30	20	4.12	
SMG	40	−66	−20	30	3.88	
SMG	40	−66	−32	22	3.37	
S1						
PoG	1	−64	−16	24	3.75	
Pu						
Pu	/	−28	2	−8	4.28	282
Pu	/	−30	10	2	3.78	
INS						
AIC	13	−44	8	−4	3.47	
AIC	13	−42	4	−2	3.44	
C. MA>LA						
No significant clusters						
D. LA>HA						
SMA						
PrG	6	46	−18	64	4.49	221
SMC	6	38	−18	60	4.15	
S1						
PoG	1	52	−16	52	3.61	
E. MA>HA						
No significant clusters						
F. LA>MA						
No significant clusters						

SMG: supramarginal gyrus; PoG: postcentral gyrus; AIC: anterior insula cortex; INS: insula; COp: central operculum; SMC: supplementary motor cortex; PrG: precentral gyrus; IFGOp: opercular part of the inferior frontal gyrus; IFGOr: orbital part of the inferior frontal gyrus; IFGTr: triangular part of the inferior frontal gyrus; S1: primary somatosensory cortex; GP: globus pallidus; VDC: ventral diencephalon; POp: parietal operculum; VLPFC: ventrolateral prefrontal cortex; Pu: putamen.

Supplementary Table S3 MNI coordinates of the contrasts across anticipation cues for all participants during pain anticipation. The significant activation of brain regions for all participants can be accepted with an uncorrected p-value of < 0.001 at the voxel level and a cluster-level p-value of < 0.05 corrected using FWE. BA: Brodmann's area.

**Region**	**BA**	X **[mm]**	Y **[mm]**	Z **[mm]**	Z**-value**	**Voxels**
A. HS/HA>HS/MA						
MTG						
AnG	39	48	−62	18	4.58	225
FuG	37	62	−54	12	3.32	
SPL						
PCun	7	−6	−46	70	4.38	999
PCun	7	−4	−44	58	3.96	
PCun	7	−14	−54	50	3.85	
dPCC						
cgm	31	−12	−38	50	4.08	
cgp	31	−8	−36	48	3.81	
cgp	31	10	−28	40	3.72	
cgp	31	4	−30	48	3.48	
SMA						
cgm	/	22	−24	40	3.81	
cgm	6	16	−24	42	3.74	
M1						
PCL	4	2	−34	66	3.46	
Cb						
Cb	/	−2	−32	−28	4.36	333
Cb	/	4	−26	−38	3.94	
Cb	/	−6	−18	−28	3.54	
Cb	/	−8	−22	−32	3.49	
Cb	/	4	−20	−28	3.41	
VC						
MOG	19	−44	−78	26	4.26	354
MTG	39	−50	−70	18	4.15	
A1						
STG	41	−46	−26	8	4.08	222
STG	41	−50	−16	6	3.21	
B. LS/MA>LS/LA						
Cd						
Cd	/	−22	−20	24	5.12	291
Cd	/	−22	−8	26	4.17	
VLPFC						
IFGOp	44	54	20	34	4.70	352
DLPFC						
MFG	9	42	34	28	4.33	
MFG	9	36	28	18	3.94	
LV						
LV	/	10	−4	20	4.51	504
LV	/	8	−18	22	3.35	
Cd						
Cd	/	18	−12	24	4.46	
Cd	/	16	8	20	3.36	
Thalamus						
Thalamus	/	8	−2	8	4.09	
SMA						
SMC	/	0	16	54	4.33	261
SFGM	8	8	30	48	4.24	
SFG	8	8	30	48	4.24	
SFG	6	12	22	58	3.43	
SFG	6	16	24	62	3.14	

continued on next page

continued from previous page

**Region**	**BA**	*X* [mm]	*Y* [mm]	*Z* [mm]	*Z*-value	**Voxels**
C. HS/MA>LS/MA						
No significant clusters						
D. HS/MA>HS/HA						
No significant clusters						
E. LS/LA>LS/MA						
No significant clusters						
F. LS/MA>HS/MA						
SMA/M1/PCun/	6/4/7/					
SPL/S1/ACC	31/1/32	−4	−18	58	5.81	5266
FEF/DLPFC/SMA	8/9/6	2	28	38	4.84	562
SMG	40	50	−38	26	4.53	328
STG/SMG/A1	22/40/41	−56	−32	8	4.48	680
MOG/IOG/	19/18/					
AnG/PCun/FuG	39/31/37	−56	−32	8	4.48	1699
STG/A1/Hi	22/41/54	46	−10	−10	4.23	420
MOG/IOG	18/19	−32	−90	4	4.20	611
STG	22	52	−40	10	4.15	245
Pu/Cd/SCA/OlfA	49/48/25/24	8	28	−8	4.12	299
M1	4	56	−10	32	4.09	228
DLPFC/MFG/INS/VLPFC	9/8/45/10	−36	22	30	4.06	333

SMG: supramarginal gyrus; PCun: precuneus; SMC: supplementary motor cortex; SPL: superior parietal lobule; IOG: inferior occipital gyrus; FuG: fusiform gyrus; MTG: middle temporal gyrus; SFG: superior frontal gyrus; PrG: precentral gyrus; MFG: middle frontal gyrus; IFGOp: opercular part of the inferior frontal gyrus; dPCC: dorsal posterior cingulate cortex; cgm: middle segment of cingulum; cgp: posterior cingulate gyrus; M1: primary motor cortex; PCL: paracentral lobule; Cb: cerebellum; VC: visual cortex; MOG: middle occipital gyrus; A1: primary auditory cortex; Cd: caudate nucleus; LV: lateral ventricle; SFGM: superior frontal gyrus medial segment; PrGM: precentral gyrus medial segment; FEF: frontal eye field; Hi: hippocampus; Pu: putamen; SCA: subcallosal area; OlfA: olfactory area; IFGTr: triangular part of the inferior frontal gyrus; VLPFC: ventrolateral prefrontal cortex.

Supplementary Table S4 MNI coordinates of the activated clusters in temporal causality analysis of the peripheral and cerebral hemodynamic responses during pain anticipation. The significant activation of brain regions for all participants can be accepted with an uncorrected p-value of < 0.001 at the voxel level and a cluster-level p-value of < 0.05 corrected using FWE. BA: Brodmann's area.

**Region**	**BA**	X **[mm]**	Y **[mm]**	Z **[mm]**	Z**-value**	**Voxels**
A. Lag $t_d = -3$ (s)						
No significant clusters						
B. Lag $t_d = -2$ (s)						
V2						
IOG	18	−28	−96	−4	5.18	294
VLPFC						
FOp	45	40	22	8	4.31	356
INS						
AIC	13	32	26	6	3.98	
C. Lag $t_d = -1$ (s)						
V2						
IOG	18	−30	−96	−4	5.27	276
V2						
IOG	18	32	−92	−8	4.89	290
INS						
AIC	13	36	14	−6	4.06	502
IFGOr	13	34	28	−2	3.68	
VLPFC						
FOp	44	44	16	4	3.81	
ACC						
ACgG	32	6	34	12	4.18	431
ACgG	24	−6	34	16	4.14	
DLPFC						
SMC	8	−4	24	46	4.04	345
SMC	8	10	20	36	3.49	
D. Lag $t_d = 0$ (s)						
VLPFC						
FOp	44	42	10	8	4.14	234
IFGTr	47	44	30	0	3.76	
INS						
AIC	13	36	16	2	3.36	
MCC						
MCgG	24	2	22	24	4.08	751
DLPFC						
SMC	8	4	20	44	3.77	
V2						
IOG	18	32	−92	−6	3.98	299
E. Lag $t_d = +1$ (s)						
V2						
IOG	18	32	−92	−6	3.74	201
OcP	18	28	−96	0	3.59	
F. Lag $t_d = +2$ (s)						
No significant clusters						
G. Lag $t_d = +3$ (s)						
No significant clusters						

SPL: superior parietal lobule; PCun: precuneus; PoG: postcentral gyrus; IOG: inferior occipital gyrus; FOp: frontal operculum; AIC: anterior insula cortex; IFGOr: orbital part of the inferior frontal gyrus; ACgG: anterior cingulate gyrus; SMC: supplementary motor cortex; IFGTr: triangular part of the inferior frontal gyrus; MCgG: middle cingulate gyrus; OcP: occipital pole.

Supplementary Table S5 MNI coordinates of the activated clusters in temporal causality analysis of the peripheral and cerebral hemodynamic responses during pain perception. The significant activation of brain regions for all participants can be accepted with an uncorrected p-value of < 0.001 at the voxel level and a cluster-level p-value of < 0.05 corrected using FWE. BA: Brodmann's area.

**Region**	**BA**	X**[mm]**	Y**[mm]**	Z**[mm]**	Z**-value**	**Voxels**
A. Lag $t_d = -3$ (s)						
No significant clusters						
B. Lag $t_d = -2$ (s)						
INS						
AIC	13	32	20	8	4.13	224
VLPFC						
COp	44	38	8	8	3.47	
FOp	44	44	16	2	3.36	
C. Lag $t_d = -1$ (s)						
S1						
PoG	2	54	−22	28	3.71	236
SMG						
SMG	40	64	−30	26	3.68	
D. Lag $t_d = 0$ (s)						
SMG						
SMG	40	62	−28	30	3.59	179
E. Lag $t_d = +1$ (s)						
No significant clusters						
F. Lag $t_d = +2$ (s)						
No significant clusters						
G. Lag $t_d = +3$ (s)						
No significant clusters						

SMG: supramarginal gyrus; PoG: postcentral gyrus; AIC: anterior insula cortex; COp: central operculum; FOp: frontal operculum; PCun: precuneus; OcP: occipital pole; SMC: supplementary motor cortex; SPL: superior parietal lobule; IOG: inferior occipital gyrus; OFuG: occipital fusiform gyrus; FuG: fusiform gyrus; MTG: middle temporal gyrus; MCgG: middle cingulate gyrus; SFG: superior frontal gyrus; TMP: temporal pole; PrG: precentral gyrus; MFG: middle frontal gyrus; PCgG: posterior cingulate gyrus; IFGOp: opercular part of the inferior frontal gyrus.
